# Contrasted Effects of Diversity and Immigration on Ecological Insurance in Marine Bacterioplankton Communities

**DOI:** 10.1371/journal.pone.0037620

**Published:** 2012-06-12

**Authors:** Thierry Bouvier, Patrick Venail, Thomas Pommier, Corinne Bouvier, Claire Barbera, Nicolas Mouquet

**Affiliations:** 1 Laboratoire d’Ecologie des Systèmes Marins Côtiers (ECOSYM), Université Montpellier 2, UMR5119, CNRS IRD IFREMER, Place E. Bataillon, Montpellier, France; 2 Institut des Sciences de l’Evolution (ISEM), UMR 5554, CNRS, Université Montpellier 2, Place E. Bataillon, Montpellier, France; 3 School of Natural Resources and Environment, University of Michigan, Ann Arbor, Michigan, United States of America; 4 Université de Lyon, UMR 5557 Ecologie Microbienne (CNRS, Université Lyon 1, USC INRA 1193), bat. G. Mendel, Villeurbanne, France; National Institute of Water & Atmospheric Research, New Zealand

## Abstract

The ecological insurance hypothesis predicts a positive effect of species richness on ecosystem functioning in a variable environment. This effect stems from temporal and spatial complementarity among species within metacommunities coupled with optimal levels of dispersal. Despite its importance in the context of global change by human activities, empirical evidence for ecological insurance remains scarce and controversial. Here we use natural aquatic bacterial communities to explore some of the predictions of the spatial and temporal aspects of the ecological insurance hypothesis. Addressing ecological insurance with bacterioplankton is of strong relevance given their central role in fundamental ecosystem processes. Our experimental set up consisted of water and bacterioplankton communities from two contrasting coastal lagoons. In order to mimic environmental fluctuations, the bacterioplankton community from one lagoon was successively transferred between tanks containing water from each of the two lagoons. We manipulated initial bacterial diversity for experimental communities and immigration during the experiment. We found that the abundance and production of bacterioplankton communities was higher and more stable (lower temporal variance) for treatments with high initial bacterial diversity. Immigration was only marginally beneficial to bacterial communities, probably because microbial communities operate at different time scales compared to the frequency of perturbation selected in this study, and of their intrinsic high physiologic plasticity. Such local “physiological insurance” may have a strong significance for the maintenance of bacterial abundance and production in the face of environmental perturbations.

## Introduction

Marine bacterial communities are recognized as major players in the ecology of coastal ecosystems, i.e. nutrient cycling, production and decomposition of organic matter, and the regulation of major biogeochemical cycles [1], [2]. Yet, they have only recently been incorporated into the research agenda regarding the relationship between biological diversity and ecosystem functioning [3], [4]. Empirical evidence of how aquatic bacterial diversity affects ecosystem functioning in the field is still equivocal and neither the intensity nor the outcome of their effect is well understood [5].

Marine bacterioplankton consist of drifting microorganisms that often inhabit large volumes of water. This particularity necessitates the inclusion of their spatial distribution as a central element in understanding the relationship between their diversity and ecosystem functioning. Indeed, while it is intuitive that in connected aquatic systems “everything is everywhere” [6], [7], it is now acknowledged that patterns of diversity exist over small and large spatial scales [5], [8], [9] and that immigration may be a key feature in explaining the assemblage of bacterial communities [10]–[13]. Adding a spatial perspective to bacterioplankton community assembly permits the consideration of these systems as potentially organized in metacommunities [14], i.e. a regional set of local communities connected by migration [10]. This concept holds that species coexistence, at both local and regional scales, is influenced by the interaction of migration between local communities and competition within local communities. This theory provides new insights into how communities are structured at multiple spatial scales; in particular on the relationship between species richness and ecosystems functioning. For example, recently, Lindström and Östman [15] reported contrasting experimental effects of dispersal on bacterioplankton metacommunity functioning.

In a temporally fluctuating environment, diversity may increase the stability of a community through different mechanisms [16]–[19]. Among these mechanisms, the insurance hypothesis [20] proposes that species richness can act as a buffer for ecosystem functioning (i.e., reduction in temporal variance). This effect is mediated by asynchronous and compensatory responses of species to environmental fluctuations. The spatial insurance hypothesis [21] applies this idea in a metacommunity context, considering that species compensation against temporal variation arises from spatial environmental complementarity between species and migration among communities. Overall, the insurance hypothesis predicts an increase in the temporal mean of ecosystem productivity (performance enhancing effect) and a decrease in its temporal variability (buffering effect) in more species rich communities. Migration between local communities will maximize the insurance effect of species diversity in metacommunites [21]. Despite clear theoretical predictions, empirical evidence for the effect of dispersal on stability remains scarce and controversial [22]–[25]. Given the central role of aquatic bacteria in ecosystem functioning [1], the insurance hypothesis, if applicable to marine bacterioplankton, should have considerable implications for current threats on diversity associated with human activities and global change.

We investigated how coastal bacterial assemblages with different initial levels of diversity and immigration responded to temporal environmental variation. We used bacterial communities from two distinct Mediterranean lagoons, the Thau and Bagnas lagoons. These two lagoons differ in their salinity, chlorophyll *a* concentrations, and in their bacterial community structure. Bacterioplankton communities were transferred between lagoons to mimic changes in environmental conditions experienced by natural aquatic bacterial assemblages [26]. The Thau lagoon bacterioplankton community was transferred from Thau lagoon water (native environment) to Bagnas lagoon water (foreign environment) before being returned back to Thau lagoon water. This alternating transfer was performed twice in 10 days. By manipulating immigration into each bacterioplankton community, following transfer, we could simulate a metacommunity comprising water and bacterioplankton communities from the two lagoons. We found that under a fluctuating environment, initial diversity and levels of immigration in to the bacterioplankton community had contrasting effects on key bacterial attributes, such as cell abundance and production. We discuss these results within the context of ecological insurance and bacterial physiological particularities.

## Methods

Testing the insurance hypothesis with natural bacterioplankton communities requires the whole community to be subjected to a temporally fluctuating environment. This was achieved by successive transfer of bacterioplankton lagoon communities, with differing levels of diversity and immigration over two contrasting environments.

### Sites and Experimental Approach

**Figure 1:**
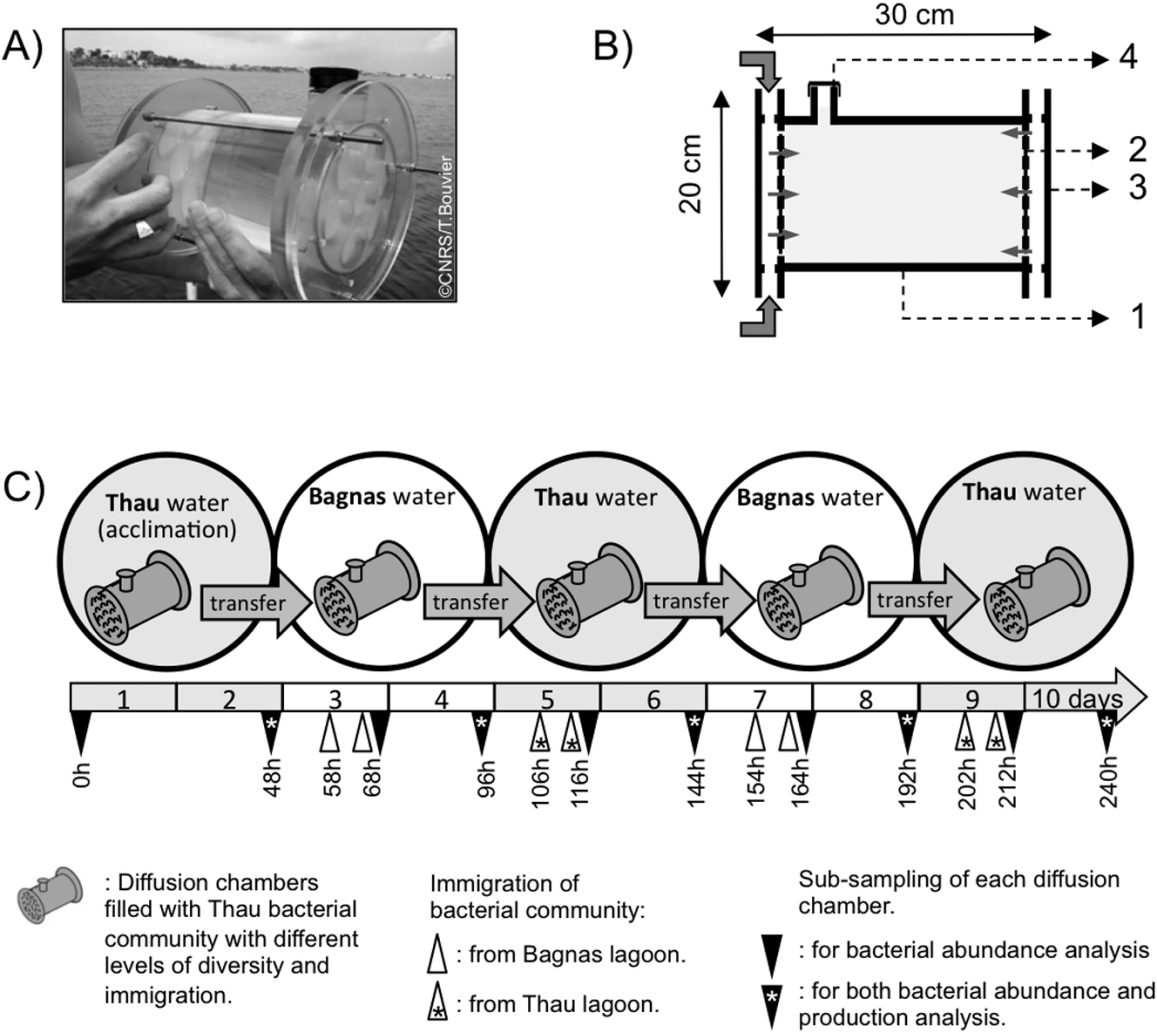
Illustration of the diffusion chambers and experimental design of the experiment carried out in this study. A) The 2-L capacity diffusion chambers used in this study. B) A diffusion chamber consists of a 120 mm diameter Plexiglas® cylinder (1), with 0.22 µm pore size polycarbonate membranes at both ends (2) allowing ample percolation of water and dissolved substances (grey arrows). The thin membrane is protected by a Plexiglas protection (3). Sampling was done by opening the cap of the chamber (4). Rubber seals were used for water-tightness. C) The bacterial community from the Thau lagoon was incubated within the diffusion chambers. After a 48 h period of acclimation in Thau lagoon water, the bacterioplankton were transferred successively in their chambers between the Bagnas water tank and the Thau water tank every 48 hours. This resulted in bacterial communities experiencing each environment twice. Each chamber was sampled nine times during the experiment (black triangles). D) Three different levels of diversity and dispersal rates were tested (High, Medium, and Low diversity, and 0%, 1% and 10% of immigration, respectively; see Methods). All treatments were replicated three times. Chambers with 1% and 10% immigration received immigration twice (10 h and 20 h) after each transfer (white triangles).

The 50 km wide coastal area near Montpellier, in the south of France, includes a variety of lagoons and ponds with different environmental conditions (anoxic/oxic conditions, freshwater/seawater ratio, eutrophic/oligotrophic levels), most of which are connected to the Mediterranean Sea [27]. On the first day of the experiment, we collected 1000 L of water from two lagoons, with contrasting salinity and Chlorophyll *a* concentrations (Chl *a*), over a short geographic distance (<10 km): the Bagnas (43°24′53′′N - 3°41′16′′E) and the Thau (43°19′47′′N - 3°31′13′′E) lagoons. Salinity measured with a Cond probe 197i were 8 and 34 respectively, and Chl *a* concentrations (fluorimetric measurements, [28]) were 0.4 and 11 µg l^−1^ Chl *a* in Bagnas and Thau respectively. Collected water was stored in 100 L plastic drums, that had been acid-washed (24 h in 10% HCl) and rinsed in deionized water, and were immediately transferred to a field laboratory located at the edge of Thau lagoon. We filled two 900-L polypropylene tanks with either 1 mm filtered water from the Thau or the Bagnas lagoons and allowed a constant water flow (1 day retention time). Our experimental approach consisted of transferring the Thau bacterial community between tanks containing water from the Thau and the Bagnas lagoons to experimentally mimic temporal variation in environmental conditions (Fig. 1). In practice, the Thau bacterial community was incubated in 2-L diffusion chambers that were separated from the surrounding water (Thau or Bagnas) by two 0.22-µm-pore-size polycarbonate membranes, Whatman® (Fig. 1A, B). These membranes are permeable to molecules such as nutrients and salts but are impermeable to most bacterial cells. This system allows bacterial communities to be monitored while soluble products can diffuse across membranes.

### Incubations in a Variable Environment and Sampling

Prior to filling with bacterioplankton communities, diffusion chambers were acid-washed (24 h in 10% HCl) and rinsed thrice in deionized water and dried. Chambers were filled with bacterioplankton communities from the Thau Lagoon with three different levels of diversity (see below). Figure 1C illustrates the experimental design. Briefly, for the first 48 hours of the experiment, the diffusion chambers were incubated in the 900-L tank filled with water from the Thau lagoon. After this acclimation period, the chambers were transferred four times between the Thau and Bagnas water tanks. For the first transfer, all the chambers were transferred into the 900-L tank filled with water from the Bagnas lagoon and incubated there for 48 hours. For the second transfer, the chambers were transferred back to into the tank with water from the Thau lagoon and incubated 48 hours. The third and fourth transfers consisted in incubating the chambers for 48 hours in the Bagnas and Thau water tanks respectively. Between each transfer, the water in each tank was replaced by fresh lagoon water collected following the same procedure as described above. The experiment was done in the dark to avoid phytoplankton growth. Our experimental setup intended to create a realistic environmental change that affects bacterioplankton communities. The probability of a salinity difference of 26 over a 2 days period is realistic and likely to be a factor influencing community structure [27], [29].

**Figure 2:**
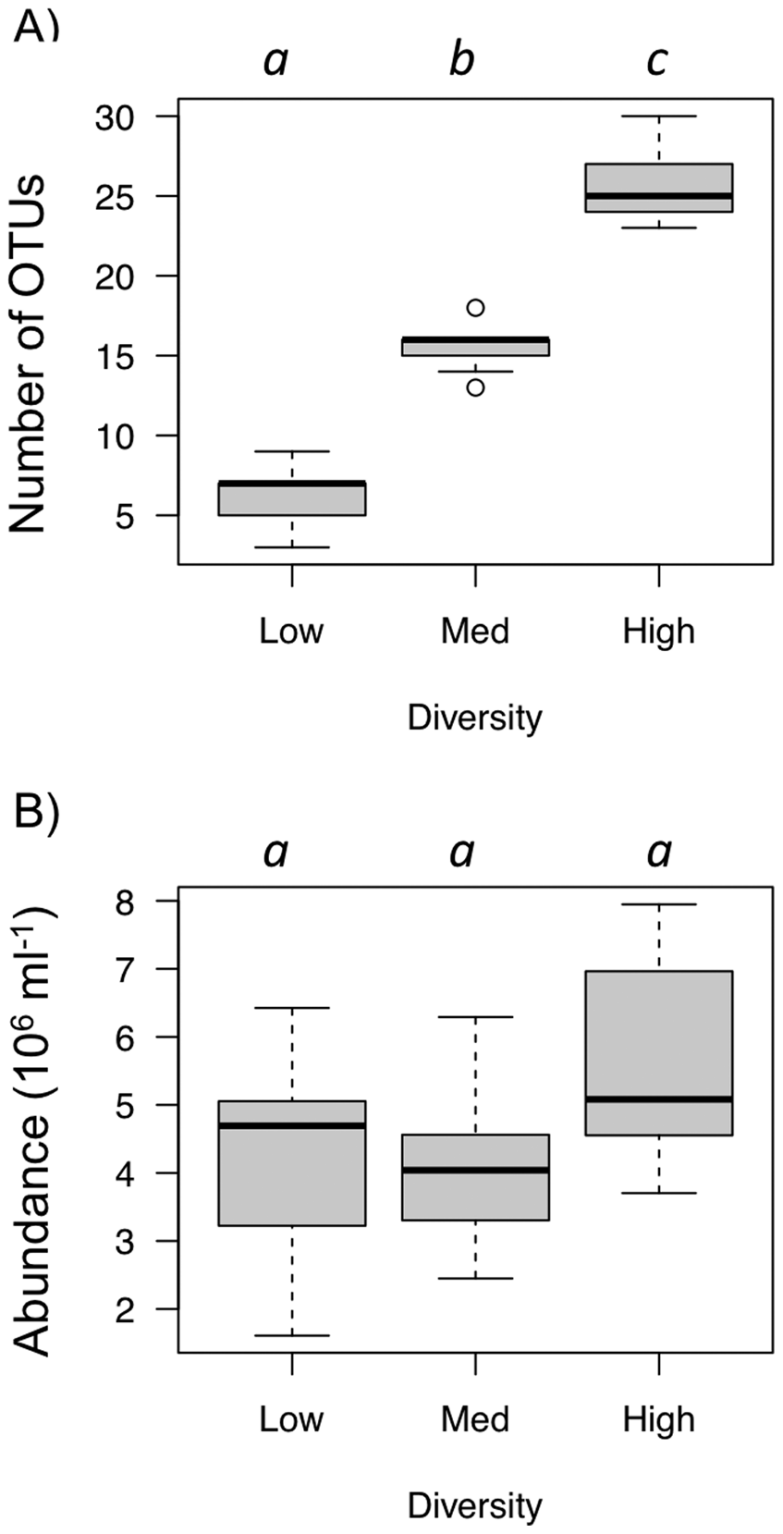
Preliminary tests results for initial bacterial diversity gradient and abundances. (A) Number of operational taxonomic units (OTUs) for the three bacterial diversity levels after the acclimation period (48 h of incubation in Thau lagoon water, Fig. 1C). Per diversity level, the three immigration treatments are combined (no immigration, 1% and 10% of immigration, n = 9). Dilution rates are: low = 10^−5^, Med = 10^−3^ and High = no dilution (see methods). (B) Bacterial abundance after the acclimation period for the three diversity treatments. The three immigration treatments are combined per diversity level. Diversity levels connected by the same letter are not significantly different (Tukey-Kramer test, *p*<0.05).

### Diversity and Immigration Treatments

The dilution-to-extinction approach was used to obtain three different starting levels of bacterioplankton diversity. Dilution removes the rare species and thereby different dilutions create communities that differ in their diversity. This approach allowed us to explore the link between community diversity and functioning [30]. Three dilutions for the Thau bacterioplankton community were chosen based on formerly established levels of bacterial diversity; hereafter called “high” (no dilution), “medium” (dilution 10^−3^) and “low” (dilution 10^−5^) diversity. Measuring band number in a denaturing gradient gel electrophoresis (DGGE, see below for methods and preliminary tests) confirmed that a fraction of the community had been eliminated. As DGGE is effective for detecting dominant bacterial operational taxonomic units (OTU), a decrease in DGGE band number between two dilutions is consistent with a reduction in community diversity. Dilutions of bulk bacterial communities were done with sterile lagoon water (0.22 µm filtrated plus two autoclave cycles at 121°C for 20 min). The dilution treatment could itself influence initial abundances within incubated communities. We thus controlled for bacterial abundances by allowing a 48 h acclimation period in Thau lagoon water before the first environmental transfer.

**Figure 3:**
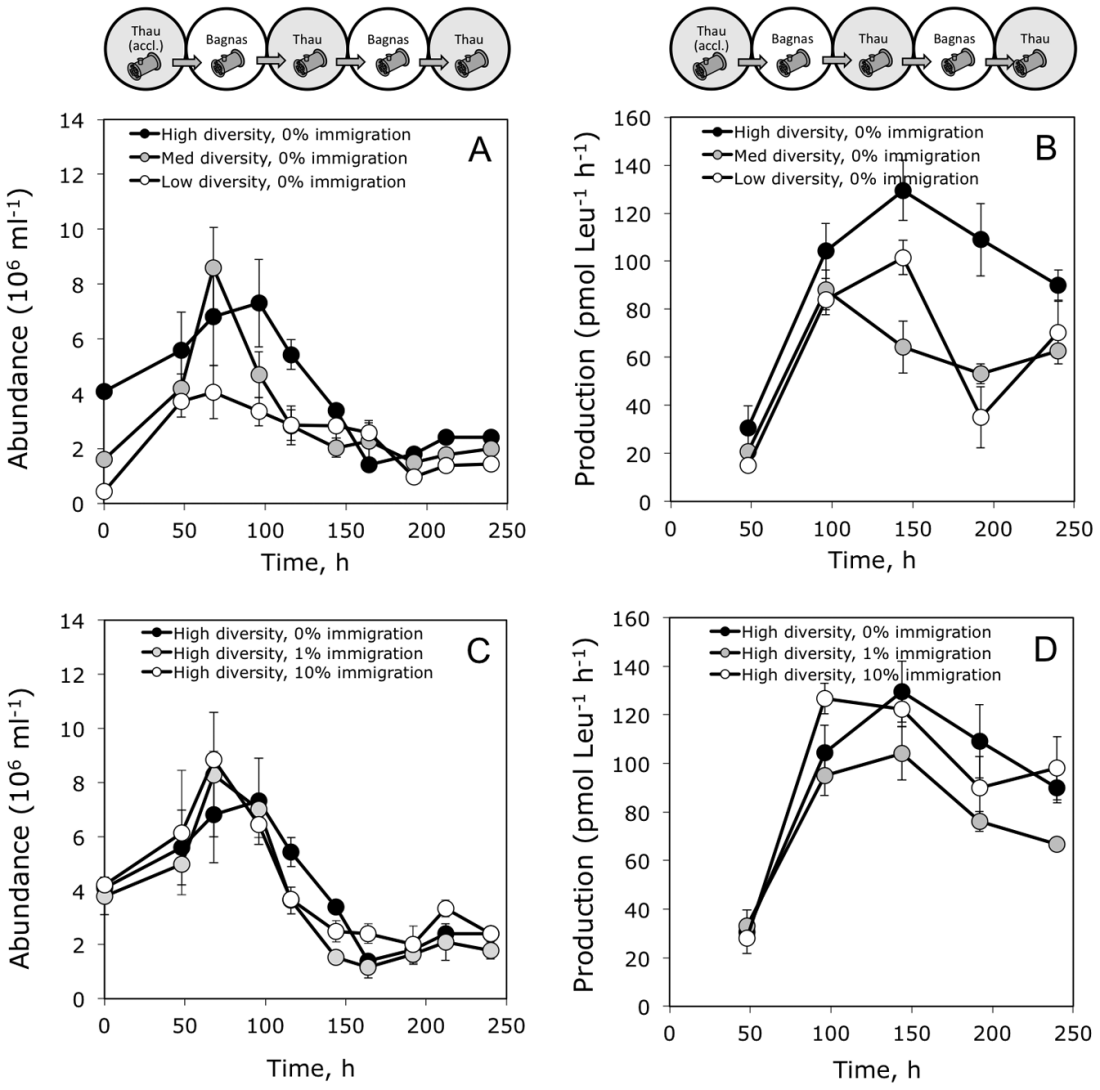
Bacterial growth within the diffusion chambers during the experiment. Bacterial abundance and production within chambers with (A, B) different levels of initial bacterioplankton diversity, in the absence of immigration, and with (C, D) different levels of bacterioplankton immigration at the high diversity level. The sketch above A and B is a reminder of the serial transfers between environments during the experiment (detailed in Fig 1). Error bars represent the standard deviation for three replicate chambers within each treatments.

**Table 1:** ANOVA’s for the effects of time, diversity and immigration on bacterial abundance and production during the experiment.

	Bacterial abundance		Bacterial production	
Effect	F	p-value	F	p-value
Time	91.67	<0.0001	11.427	0.001
Diversity	5.08	0.0075	8.85	0.0003
Immigration	1.64	0.198	0.145	0.865
Diversity*	4.962	0.0084	8.886	0.0002
Immigration*	1.602	0.206	0.145	0.865
Diversity × immigration*	0.871	0.484	0.8814	0.477

* Effects tested on a separate ANOVA on the residuals after eliminating the effect of time.

**Figure 4:**
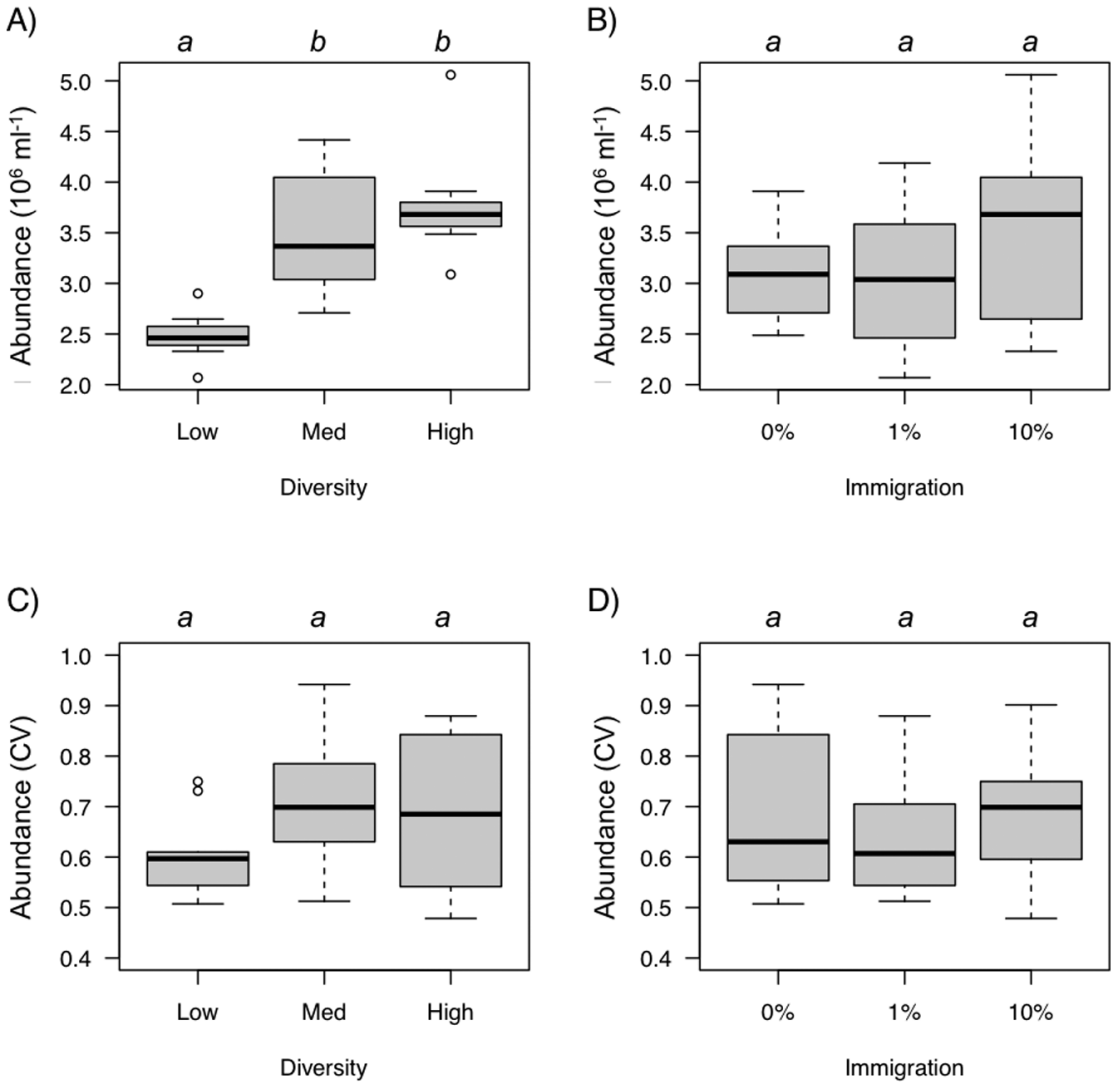
The effect of diversity (A) and immigration (B) on global temporal mean bacterial abundance during the experiment (from 48 h to 240 h of incubation), and the effect of diversity (C) and immigration (D) on the temporal coefficient of variation (CV) for bacterial abundance. Diversity or immigration levels connected by the same letter are not significantly different (Tukey-Kramer test, *p*<0.05).

**Table 2:** ANOVA’s for the effects of diversity and immigration on the temporal CV for abundance and production during the experiment.

	CV bacterial abundance		CV bacterial production	
Effect	F	p-value	F	p-value
Diversity	1.777	0.198	17.527	<0.0001
Immigration	0.464	0.635	1.039	0.374
Diversity × immigration	3.067	0.0432	2.448	0.084

Identical series of initial diversity levels were prepared for each of the three immigration treatments: no immigration, medium (1% of total abundance) and high (10% of total abundance) immigration. Immigration events were performed twice for each chamber, at each transfer (10 h and 20 h after the chambers had been transferred into the new environments, Fig. 1C), for a total of 8 immigration events during the experiment. The migrants originated from the same communities as the environment to which the chambers were exposed (e.g. chambers in Bagnas water received bacterial immigrants from the Bagnas lagoon). We chose to scale the diversity level of the immigrating population to the corresponding diversity treatments. We crossed initial and immigration diversity treatments resulting in 9 different scenarios that were replicated three times resulting in 27 chambers in total (3 diversity × 3 immigration × 3 replicates). During the incubation period, 10 sub-samples for bacterial abundances and production measurements were collected: One at the beginning of the incubation period, another after 48 h of acclimation, and then 8 sub-samples were collected after the first 20 h of incubation in the transplanted water as well as before each transfer (Fig. 1C). When immigration and sub-sampling occurred at the same time (after 20 h of incubation in the transplanted water), sub-sampling was always done prior to immigration.

**Figure 5:**
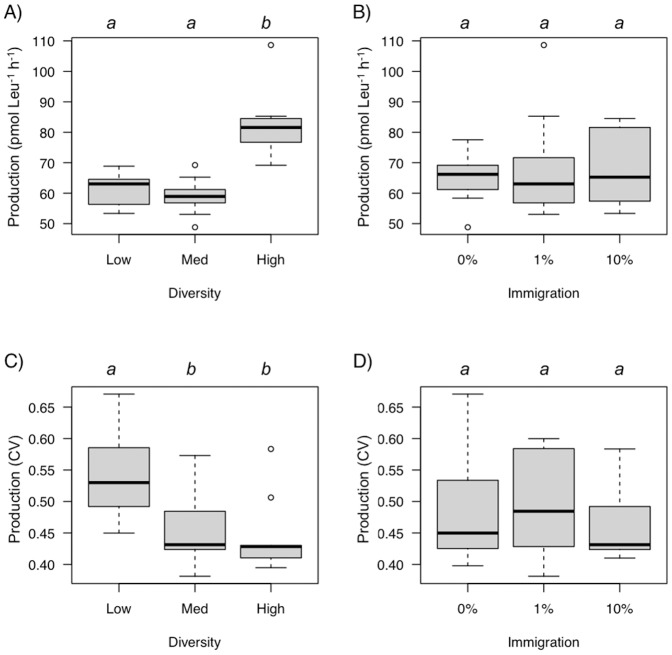
The effect of diversity (A) and immigration (B) on global temporal mean bacterial production during the experiment (from 48 h to 240 h of incubation) and the effect of diversity (C) and immigration (D) on the temporal coefficient of variation (CV) for production. Diversity or immigration levels connected by the same letter are not significantly different (Tukey-Kramer test, *p*<0.05).

### Bacterial Community Abundance and Production

Total bacterial abundance was obtained from 1 ml paraformaldehyde fixed sub-samples (1% final concentration) using a FACSCalibur flow cytometer (BectonDickinson) after nucleic acid staining with green fluorescent SYBR®GreenI (Invitrogen). Light scattering was used in conjunction with fluorescence to discriminate bacterial cells [31]. Stained bacteria were discriminated on the basis of their fluorescence and light scattering. Rates of bacterial production were measured on 2 ml of fresh subsamples from the incorporation of ^3^H-leucine following the centrifugation method of Smith and Azam [32]. Bacterial abundance and production was expressed in cell ml^−1^, and pmol Leu l^−1^ h^−1^, respectively. Bacterial abundance was measured for all sub-samples and bacterial production was measured for five sub-samples (fig. 1C).

### Bacterial Community Structure

The initial bacterial community diversity was assessed by denaturing gradient gel electrophoresis (DGGE) analysis of the 16S rRNA genes after touchdown PCR amplification [33], [34]. Pre-filtered (3 µm, Whatman) 50 ml subsamples were filtered on 0.22 µm polycarbonate filters (Whatman) and kept at −20°C until further analysis. Nucleic acid extraction from the filter was done following Boström and collaborators [35]. The V3 region of 16S rRNA genes from bacterial communities was amplified by PCR using two primers, 338f-GC and 518r. PCR was done using PuRe Taq® Ready-To-Go® PCR beads (GE Healthcare), in a Mastercycler®ep (Eppendorf). DGGE was performed with the DCode® system (Bio-Rad). PCR samples were loaded onto 8% (wt/vol) polyacrylamide gels made with a denaturing gradient ranging from 40% to 60% (100% denaturant contains 7M urea and 40% formamide). Electrophoresis was performed in 0,5X TAE buffer (Euromedex) at 60°C at a constant voltage of 100 V for 18 h. The gels were then stained for 10 min with 3 µL of 10 000X SYBR® Green I (Molecular Probes) diluted in 30 mL 0.5X TAE. DGGE banding patterns were visualized on an UV transillumination table with the imaging system GelDoc® XR (Bio-Rad). We considered DGGE bands as bacterial operational taxonomic units (OTUs), which were considered representative of predominant bacterial “species” [36]. Resulting profiles were normalized to the bands ladder.

### Data Analyses

Time series for bacterial abundance and production were first analyzed by a three factor analysis of variance (ANOVA) with time, diversity and immigration included as explanatory variables. No interactions among factors were included. Then, we ran a second fully factorial ANOVA on the residuals (thus taking account of the effect of time) with diversity and immigration as explanatory factors. This reveals the effect of diversity and immigration on the abundance and production of our bacterial communities independently of the effect of time. We performed Tukey-Kramer post-hoc tests (alpha level  = 0.05) to test for differences between diversity and immigration levels on temporal averaged abundance and production.

We also calculated the temporal coefficient of variation (CV) for both bacterial abundance and production, for each replicate population, from 48 h (i.e. after acclimation) to the end of the experiment (240 h). The coefficient of variation is a common and convenient metric for temporal stability [37] as demonstrated by its use in more than 50 empirical studies as a measure of temporal stability across a variety of systems [17], [19]. We used a fully factorial analysis of variance (ANOVA) and Tukey-Kramer means post-hoc comparison tests (alpha level  = 0.05) to identify the precise effects of different diversity and immigration levels on temporal CVs. We assessed similarity between DGGE banding patterns from both in situ Bagnas and Thau lagoons bacterioplankton communities using the Sørensen-Dice coefficient.

### Preliminary Tests

#### Diffusion rate through the chambers

The diffusion rate of the solutes through the 0.22 µm chamber membranes must be greater than the incubation time between two transfers (48 h) so that communities experience changes in their environment. When chambers containing Thau water were immersed in Bagnas water, the changes in salinity inside the diffusion chambers were gradual and reached equilibrium after 15 h of incubation (Fig. S1). Clogging of membranes due to bacterial development reduces the diffusion rate marginally (salinity reaching equilibrium after 19 h of incubation, Fig. S1). These measures confirm that the Thau bacterioplankton communities experienced changes in its environment within the 48 h period of each transfer.

#### Identification of possible contamination within the diffusion chamber

Lagoon bacterial cells did not contaminate the experimental bacterial communities within each chamber during a 10 days submersion period (Fig. S2). This confirms that our results were not biased by cell addition from the surrounding water.

#### In situ bacterioplankton DGGE banding pattern from both Bagnas and Thau lagoons

A pre-requisite of the experiment is that bacterioplankton from both lagoons differed in their bacterial community composition. One week before the experiment, triplicate water samples were collected from the Bagnas and Thau lagoons to explore differences in the bacterioplankton composition based on DGGE banding pattern analysis. Both lagoons had a similar number of OTUs, but they differed in their OTUs composition (18.3% of similarity, Fig. S3). Thus we consider the two communities to be different.

#### Diversity treatment

The DGGE analysis of the diluted Thau bacterial community confirmed that our dilution treatments resulted in a bacterial diversity gradient (Fig. 2A, *F* *_2, 24_* = 216.7, *p*<0.001).

#### Bacterial abundance before the first transplant

We found that bacterial abundances did not differ between the three diversity treatments after the 48 h acclimation period in Thau water (*F_2, 9_* = 2.8, *p = *0.140; Fig. 2B).

## Results

### Bacterial Abundances

Overall, bacterial abundances increased during the first 100 h and then decreased through time (Fig. 3). We found a strong effect of initial diversity on bacterial abundances (Table 1), with higher abundances at intermediate and high diversity levels (Fig. 4A). Immigration did not affect bacterial abundances and the diversity by immigration interaction was not significant either (Table 1, Fig. 3B). These patterns remained consistent after accounting for the effect of time (Table 1). Initial diversity and immigration did not affect the temporal CV for bacterial abundances (i.e., the inverse of abundance stability, Table 2, Fig. 4C). The diversity by immigration interaction was significant (Table 2). This interaction was driven by a significant effect of diversity on the CV (low CV at high diversity) found only when immigration was high (*F_2, 6_* = 6.88, *p = *0.028).

### Bacterial Production

Bacterial production also varied trough time (Table 1, Figs. 3B and 3D). Temporal trends in bacterial production were similar among treatments with an initial increase followed by a decrease to the end of the experiment. We found a strong effect of initial diversity on bacterial production (Table 1), with higher production at high diversity levels (Fig. 5A). Immigration did not affect bacterial production and the diversity by immigration interaction was not significant either (Table 1, Fig. 5B). These patterns remained consistent after accounting for the effect of time (Table 1). We found a significant effect of diversity on the temporal CV of production (Table 2) with higher stability (lower CV) at intermediate and high diversity levels (Fig. 5C). Immigration and the interaction with diversity had no effect on the CV of production (Table 2).

## Discussion

The insurance hypothesis predicts a buffering effect of both diversity and immigration on ecosystem functioning based on species asynchrony and compensatory dynamics. Using an experimental approach, we observed two effects of diversity when the bacterial communities are confronted with temporal environmental fluctuations. First, a *performance-enhancing effect* characterized by an increase in temporal mean bacterioplankton abundance and production, and second, a *buffering effect* characterized by a reduction in the temporal variance of production [20]. This overall positive effect of diversity on ecosystem functioning is consistent with previous results found for a wide array of ecological systems (reviewed in [38]–[40]) including microbial systems such as protists [41] aquatic, soil and biofilm bacterial communities [42], [43] and species communities associated in microbial food webs [44]. This result highlights the importance of diversity in bacterioplankton natural communities as an insurance against environmental fluctuations. This also has strong implications for ecosystem functioning as other planktonic compartments, and more generally biogeochemical cycles, are both dependent upon bacterial abundance and production rates [1].

The fundamental basis of the biological insurance lies in the temporal niche complementarity between species, and in sampling processes [20]. We have not assessed the importance of niche differentiation in our experiment. However, there is substantial literature on specific bacterial responses to environmental factors helping to delineate the niche dimensions of prokaryotes. In particular, bacterial species or phylogenetic groups do not respond equally to organic or inorganic nutrients, or to changes in physical parameters such as salinity in terms of growth and mortality [45], [46]. This may lead to the asynchrony in species responses along an environmental gradient thus permitting the insurance effect to happen. Furthermore, under new environmental conditions, specific bacteria can become dominant [47], [48] adding to the performance enhancing effect promoted by diversity insurance [20]. Disentangling the species specific contribution to the insurance effect is beyond the scope of our experiment. However, this could be investigated as a future direction through the combined use of fluorescence *in situ* hybridization with microautoradiography, or nano-scale secondary-ion mass spectrometry, which have been shown as promising tools for correlating microbial identity with specific metabolic functions, for individual cells, within heterogeneous bacterial communities (MAR-FISH and NanoSIMS, respectively; [49]). Another limitation comes from only including 2 lagoons in our study thus potentially causing a spatial confounding effect. Generalization of our findings will require a larger scale experimental set up with a higher number of lagoons and different gradients of spatial heterogeneity.

We found only a marginal effect of immigration on bacterial functioning and no effect on its temporal stability. In the spatial insurance hypothesis, source-sink dynamics among sites may rescue species from local extinction and lead to a higher level of ecosystem functioning in communities that are open to immigration than in closed communities [21]. Immigration is thus important mainly because it maintains high levels of local diversity in communities that might otherwise decline with temporal environmental fluctuations. The absence of a clear immigration effect in our experiment might have been caused by a low impact of environmental fluctuation on bacterial diversity. Indeed, the intensity of the environmental gradient might not have been high enough and/or the period of fluctuation long enough to have a strong impact on bacterioplankton. Note that our experimental design was not, in the strict sense, a true metacommunity with constant dispersal between the different communities. Rather we “simulated” metacommunity dynamics by using two pools of immigrants from each lagoon that were used for our immigration treatments. Crossed with environmental variation this allowed us to fit the assumptions of [21] (i.e. desysnchronisation of environmental variation between communities and dispersal among communities permits ecological insurance). A more complex experimental design with crossing of metacommunity topologies [14] with temporal environmental variation is required to fully test the spatial insurance hypothesis but we believe our work is a first step in this direction. The weak effect of immigration might have been due to the capacity of bacteria to survive harsh environmental conditions. Indeed, dormancy and low activity are the natural states of a significant proportion of bacteria in the aquatic biosphere [50] that resume cell division when environmental conditions change and become favorable, [51]. For instance, [52] recently demonstrated high resilience of bacterioplankton diversity to environmental perturbation. This would result in strong temporal insurance with initial diversity permitting ecological insurance, without the need of spatial insurance, as bacteria could resist prolonged periods of harsh conditions. The pattern and intensity of environmental fluctuations should thus be contrasted with the capacity of bacteria to resist and recover from periods of stress [53] to set the range of environmental conditions for spatial insurance to occur [10]. That immigration is beneficial at higher levels is consistent with recent results by Lindström and Östman [15] who found an effect of immigration on lacustrine bacterial community composition and functioning at 43% of dispersal per day. Further experiments manipulating directly the period and intensity of the environmental gradient, as well as immigration scaled to bacteria generation times are now needed. Note also that individual functional traits do not necessarily scale up to community level processes [30], [54]. Further, one may anticipate that specific individual functional traits, such as enzyme activity or transcriptional regulation of genes, not investigated in this study might have been influenced by immigration, without any detectable change in abundance or production.

Our results stress the need to remodel the paradigms used in the ecological insurance hypothesis, initially built for macroorganisms, to microorganisms. Indeed, other insurance mechanisms could be expected given the extremely high adaptive capacity of microorganisms. As we have mentioned above, dormancy, or the capacity to respond to perturbation at the level of transcription of “flexible” genes [55], [56], could be interpreted as a “physiological insurance” of the bacterial cells. Also, given the high reproduction rate of microorganisms, evolution may be fast enough to produce bacterial subpopulations with specialized functions, allowing community’s to withstand new environmental constraints (e.g. in biofilm communities, [42]). The relative importance of these different insurances for microorganisms would then be a function of the intensity and the period of environmental fluctuations. The question of whether microbes are versatile enough to insure themselves is thus central in investigating the effects of their diversity on ecosystem functioning. Future approaches will aim at identifying causal mechanisms of biological insurance in natural bacterioplankton communities coupled with ecophysiological approaches.

## Supporting Information

Figure S1 **Both the rate of diffusion through the 0.22 µm membrane of the diffusion chambers, and the effect of biofouling on that rate, were assessed using Thau lagoon water.** Two chambers were filled with bulk Thau lagoon water and immediately lowered into a 50-L capacity tank charged with the Bagnas water (Chamber #1 and #2 in figure S1). Two other chambers were filled with bulk Thau lagoon water and let 12 days in a tank charged with the Thau lagoon water to let bacteria grow. After incubation, these two last chambers have been lowered into a 50-L tank filled with the Bagnas water (Chambers #3 and #4 in figure S1). The salinity inside the chamber was measured with a Microosmometer (The advanced instruments inc.) at the time of the incubation and hourly for 24 h thereafter. Change in salinity into the chambers was gradual and reach equilibrium after approximately 15 h of incubation for chambers #1 and #2, and 19 h for chambers #3 and #4. (Fig. S1).
(DOC)

Figure S2 **We assayed how much our diffusion chambers were resistant to potential contamination from the surrounding waters in a pilot experiment performed before the main experiment.** This control of sterility was determined by incubating for 10 days four chambers (2 completely waterproof and 2 with 0.2 µm membranes) containing sterile Thau lagoon water into a flow-through natural Thau lagoon water tank. The sterile Thau lagoon water was obtained after filtration through a 0.22 µm polycarbonate membrane plus two cycles of autoclave at 121°C during 20 min. Using this procedure, bacterial abundance was abated by 98.4% (from 2.3 10^6^ cells ml^−1^ to 3.6 10^4^ cells ml^−1^). Most of the persisting cells were considered as dead cell. Two milliliters of water were withdrawn from the chambers at *t* = 0, *t* = 1 h, *t* = 24 h and *t* = 240 h. Bacterial abundance was determined by flow cytometry as described in the experimental procedures section. Bacterial abundance did not significantly change over time and between the waterproof and 0.22 µm membranes diffusion chambers (ANOVA, p>0.05; Fig. S2).
(DOC)

Figure S3 **One week before the experiment, 200-ml of water from both Thau and Bagnas lagoons were collected with a 500-ml acid washed glass bottle to determine the DGGE banding pattern of the bacterioplankton communities.** The DGGE method is describe in the experimental procedure section. The number of OTUs was similar with 17 and 20 OTUs for Thau and Bagnas bacterioplankton, respectively. However, both communities shared only 5 OTUs (Fig. S3). This results in a low similarity between the two communities (Sørensen-Dice coefficient  = 18.3%).
(DOC)
