# Supplementary material for: Contrasted Effects of Diversity and Immigration on Ecological Insurance in Marine Bacterioplankton Communities
Source: PLoS One. 2012 Jun 12;7(6):e37620. doi: 10.1371/journal.pone.0037620 (PMC3373509; doi:10.1371/journal.pone.0037620)
Supplement: Figure S1 — Both the rate of diffusion through the 0.22 µm membrane of the diffusion chambers, and the effect of biofouling on that rate, were assessed using Thau lagoon water. Two chambers were filled with bulk Thau lagoon water and immediately lowered into a 50-L capacity tank charged with the Bagnas water (Chamber #1 and #2 in figure S1). Two other chambers were filled with bulk Thau lagoon water and let 12 days in a tank charged with the Thau lagoon water to let bacteria grow. After incubation, these two last chambers have been lowered into a 50-L tank filled with the Bagnas water (Chambers #3 and #4 in figure S1). The salinity inside the chamber was measured with a Microosmometer (The advanced instruments inc.) at the time of the incubation and hourly for 24 h thereafter. Change in salinity into the chambers was gradual and reach equilibrium after approximately 15 h of incubation for chambers #1 and #2, and 19 h for chambers #3 and #4. (Fig. S1). (DOC) [file pone.0037620.s001.doc]

**Supplementary materials.**

*Bouvier et al. Contrasted effects of diversity and immigration on the biological insurance in marine bacterioplankton communities*

**Supporting information 1:**

Both the rate of diffusion through the 0.22 µm membrane of the diffusion chambers, and the effect of biofouling on that rate, were assessed using Thau lagoon water. Two chambers were filled with bulk Thau lagoon water and immediately lowered into a 50-L capacity tank charged with the Bagnas water (Chamber #1 and #2 in figure S1). Two other chambers were filled with bulk Thau lagoon water and let 12 days in a tank charged with the Thau lagoon water to let bacteria grow. After incubation, these two last chambers have been lowered into a 50-L tank filled with the Bagnas water (Chambers #3 and #4 in figure S1). The salinity inside the chamber was measured with a Microosmometer (The advanced instruments inc.) at the time of the incubation and hourly for 24h thereafter. Change in salinity into the chambers was gradual and reach equilibrium after approximately 15h of incubation for chambers #1 and #2, and 19h for chambers #3 and #4. (Fig. S1).

Figure S1:
