# Supplementary material for: Contrasted Effects of Diversity and Immigration on Ecological Insurance in Marine Bacterioplankton Communities
Source: PLoS One. 2012 Jun 12;7(6):e37620. doi: 10.1371/journal.pone.0037620 (PMC3373509; doi:10.1371/journal.pone.0037620)
Supplement: Figure S2 — We assayed how much our diffusion chambers were resistant to potential contamination from the surrounding waters in a pilot experiment performed before the main experiment. This control of sterility was determined by incubating for 10 days four chambers (2 completely waterproof and 2 with 0.2 µm membranes) containing sterile Thau lagoon water into a flow-through natural Thau lagoon water tank. The sterile Thau lagoon water was obtained after filtration through a 0.22 µm polycarbonate membrane plus two cycles of autoclave at 121°C during 20 min. Using this procedure, bacterial abundance was abated by 98.4% (from 2.3 106 cells ml−1 to 3.6 104 cells ml−1). Most of the persisting cells were considered as dead cell. Two milliliters of water were withdrawn from the chambers at t = 0, t = 1 h, t = 24 h and t = 240 h. Bacterial abundance was determined by flow cytometry as described in the experimental procedures section. Bacterial abundance did not significantly change over time and between the waterproof and 0.22 µm membranes diffusion chambers (ANOVA, p>0.05; Fig. S2). (DOC) [file pone.0037620.s002.doc]

**Supplementary materials.**

*Bouvier et al. Contrasted effects of diversity and immigration on the biological insurance in marine bacterioplankton communities*

**Supporting information 2:**

We assayed how much our diffusion chambers were resistant to potential contamination from the surrounding waters in a pilot experiment performed before the main experiment. This control of sterility was determined by incubating for 10 days four chambers (2 completely waterproof and 2 with 0.2 µm membranes) containing sterile Thau lagoon water into a flow-through natural Thau lagoon water tank. The sterile Thau lagoon water was obtained after filtration through a 0.22 µm polycarbonate membrane plus two cycles of autoclave at 121°C during 20 min. Using this procedure, bacterial abundance was abated by 98.4% (from 2.3 10^6^ cells ml^-1^ to 3.6 10^4^ cells ml^-1^). Most of the persisting cells were considered as dead cell. Two milliliters of water were withdrawn from the chambers at *t* = 0, *t* = 1h, *t* = 24h and *t* = 240h. Bacterial abundance was determined by flow cytometry as described in the experimental procedures section. Bacterial abundance did not significantly change over time and between the waterproof and 0.22µm membranes diffusion chambers (ANOVA, p>0.05; Fig. S2).


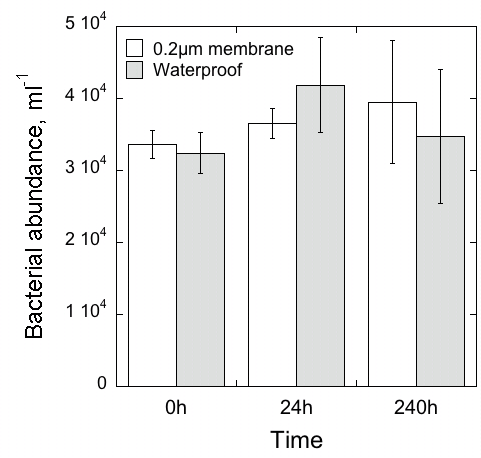


Figure S2:
