# Supplementary material for: Contrasted Effects of Diversity and Immigration on Ecological Insurance in Marine Bacterioplankton Communities
Source: PLoS One. 2012 Jun 12;7(6):e37620. doi: 10.1371/journal.pone.0037620 (PMC3373509; doi:10.1371/journal.pone.0037620)
Supplement: Figure S3 — One week before the experiment, 200-ml of water from both Thau and Bagnas lagoons were collected with a 500-ml acid washed glass bottle to determine the DGGE banding pattern of the bacterioplankton communities. The DGGE method is describe in the experimental procedure section. The number of OTUs was similar with 17 and 20 OTUs for Thau and Bagnas bacterioplankton, respectively. However, both communities shared only 5 OTUs (Fig. S3). This results in a low similarity between the two communities (Sørensen-Dice coefficient = 18.3%). (DOC) [file pone.0037620.s003.doc]

**Supplementary materials.**

*Bouvier et al. Contrasted effects of diversity and immigration on the biological insurance in marine bacterioplankton communities*

**Supporting information 3:**

One week before the experiment, 200-ml of water from both Thau and Bagnas lagoons were collected with a 500-ml acid washed glass bottle to determine the DGGE banding pattern of the bacterioplankton communities. The DGGE method is describe in the experimental procedure section. The number of OTUs was similar with 17 and 20 OTUs for Thau and Bagnas bacterioplankton, respectively. However, both communities shared only 5 OTUs (Fig. S3). This results in a low similarity between the two communities (Sørensen-Dice coefficient = 18.3%).


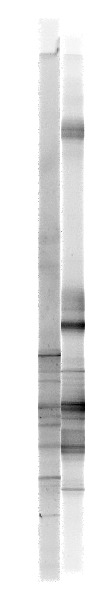
Figure S3:

03

04

05

09

10

11

13

15

17

18

19

20

22

23

26

29

32

THAU

01

02

06

07

08

11

12

14

16

18

19

21

23 24

25

27

28

29

30

31

BAGNAS
